# The effects of length and width of the stem on proximal humerus stress shielding in uncemented primary reverse total shoulder arthroplasty

**DOI:** 10.1007/s00402-023-05129-w

**Published:** 2023-11-27

**Authors:** Manuel Kramer, Martin Olach, Vilijam Zdravkovic, Melanie Manser, Patric Raiss, Bernhard Jost, Christian Spross

**Affiliations:** 1 Orthopaedic Surgery and Traumatology, Kantonsspital St. Gallen, Rorschacherstrasse 95, 9007 St. Gallen, Switzerland; 2 Orthopaedic Surgery and Traumatology, Spital Wil, SRFT, St. Gallen, Switzerland; 3 OCM (Orthopädische Chirurgie München) Clinic, Munich, Germany

**Keywords:** Cuff tear arthropathy, Omarthritis, Stress shielding, Short stem reverse total shoulder arthroplasty (RTSA), Filling ratio

## Abstract

**Introduction:**

To preserve humeral bone during RTSA, stems have been made shorter and cement avoided whenever possible. However, with the increased use of uncemented RTSA, a phenomenon comparable to the stress shielding of the hip has been described for the proximal humerus. The aim of this study was to investigate the influence of stem length and width on proximal humeral bone resorption after primary uncemented RTSA.

**Materials and methods:**

The prospective shoulder arthroplasty database of our institution was reviewed for all primary uncemented RTSAs from 2017 to 2020 in osteoarthritis and cuff tear arthropathy cases with > 2-year follow-up. We compared the clinical and the radiographic 2-year outcome of the short and standard length stems of the same prosthesis design. This allowed us to assess the effects of stem length and width with regard to stress shielding. Furthermore, we defined a cut-off value for the filling ratios to prevent stress shielding.

**Results:**

Fifty patients were included in the analysis, nineteen were in the short stem group (SHORT) and thirty-one in the standard stem group (STANDARD). After 2 years, SHORT showed a relative Constant Score of 91.8% and STANDARD of 98.3% (*p* = 0.256). Stress shielding was found in 4 patients (21%) in SHORT and in 16 patients (52%) in STANDARD (*p* = 0.03); it occurred more frequently in patients with higher humeral filling ratios (*p* < 0.05). The calculated cut-off to prevent stress shielding was 0.7 (± 0.03) for the metaphyseal and distal filling ratio.

**Conclusion:**

While short and standard stems for RTSA have good results after 2 years, we found a significant negative effect of higher length and width of the stem with regard to stress shielding. Even though the clinical effects of stress shielding have to be assessed, short stems should be chosen with a filling ratio at the metaphyseal and distal position below 0.7.

**Level of evidence (a retrospective case–control study):**

III.

## Introduction

After conservative treatments have been exhausted, reverse total shoulder arthroplasty is a good treatment option in patients with cuff tear arthropathy or patients with severely degenerative osteoarthritis of the shoulder. Although RTSA shows good long-term results, there are certain cases where revision is unavoidable [1, 2] Since the results of revision prosthetics in the shoulder joint depend on the bone stock available, any kind of bone loss should be prevented during primary implantation [3]. Uncemented stem designs are becoming more popular over the years [4], since bone could be preserved and surgical time reduced [5]. With increased use of uncemented shoulder prostheses, a stress shielding effect, well known from hip arthroplasty [6], was also observed in shoulder arthroplasty. It has already been shown that humeral stress shielding in anatomic total shoulder prostheses occurs in connection with longer and wider stems and with corresponding further distal force transmission, as well as with increased stem-to-humerus filling ratios [7–12]. So far the effect of stem length and width of a prosthesis with the same design on stress shielding has not been investigated for primary RTSA for degenerative cases. Thus, it was the primary aim of this study to fill this gap. We hypothesize that short prosthetic stems lead to less stress shielding after 2 years.

Raiss et al. defined a cut-off value of 0.8 for the distal humeral stem filling ratio in RTSA to reduce stress shielding sevenfold [13]. In this study, the width of the stem was divided by the inner cortical diameter. However, in various other studies, filling ratio measurements have already been done using the outer cortical diameter [12, 13]. There is no consensus regarding the measurement localization in the current literature. Thus, the second aim of this study was to compare these two different frequently used methods for filling ratio measurements and to compare them with regard to predicting stress shielding. We hypothesize that the measurement at the inner cortical diameter has better predictive power for stress shielding. The third aim was to define a cut-off value that may prevent stress shielding.

## Materials and methods

We performed a retrospective case–control study for total shoulder arthroplasty using standardized prospectively collected clinical evaluation data from our institution’s database. After starting with a new uncemented standard stem in 2017, a short stem version became available in 2018; it became the standard for primary non-fracture RTSA in 2019 (Medacta shoulder system: Medacta, Castel San Pietro, TI, CH). While the standard stem group included all lengths from 87 mm to 105.5 mm, all lengths from 54.1 mm to 66.5 mm belonged to the short stem group. The lengths are measured along the stem axis. Follow-up controls including radiographic and clinical data were carried out within the framework of a strict quality control system at least after 3 months, 1, 2, 5, and 10 years. Functional outcomes were assessed by a specially trained study-nurse (M.M.). This assessment included relative and absolute Constant Scores (rCS, aCS) and subjective shoulder value (SSV). Two shoulder specialists evaluated all cases for clinical or radiographic complications (B.J. and C.S.).

We used the clinical and radiographic data of all patients who underwent elective uncemented RTSA. Two groups were defined: STANDARD included cases with standard length stems implanted in 2017 and 2018; SHORT included cases with short stems implanted from 2018 to 2020. Patients with cuff tear arthropathy (CTA) or osteoarthritis (OA) of the shoulder treated with primary uncemented RTSA and a minimum follow-up of 2 years were included.

**Fig. 1:**
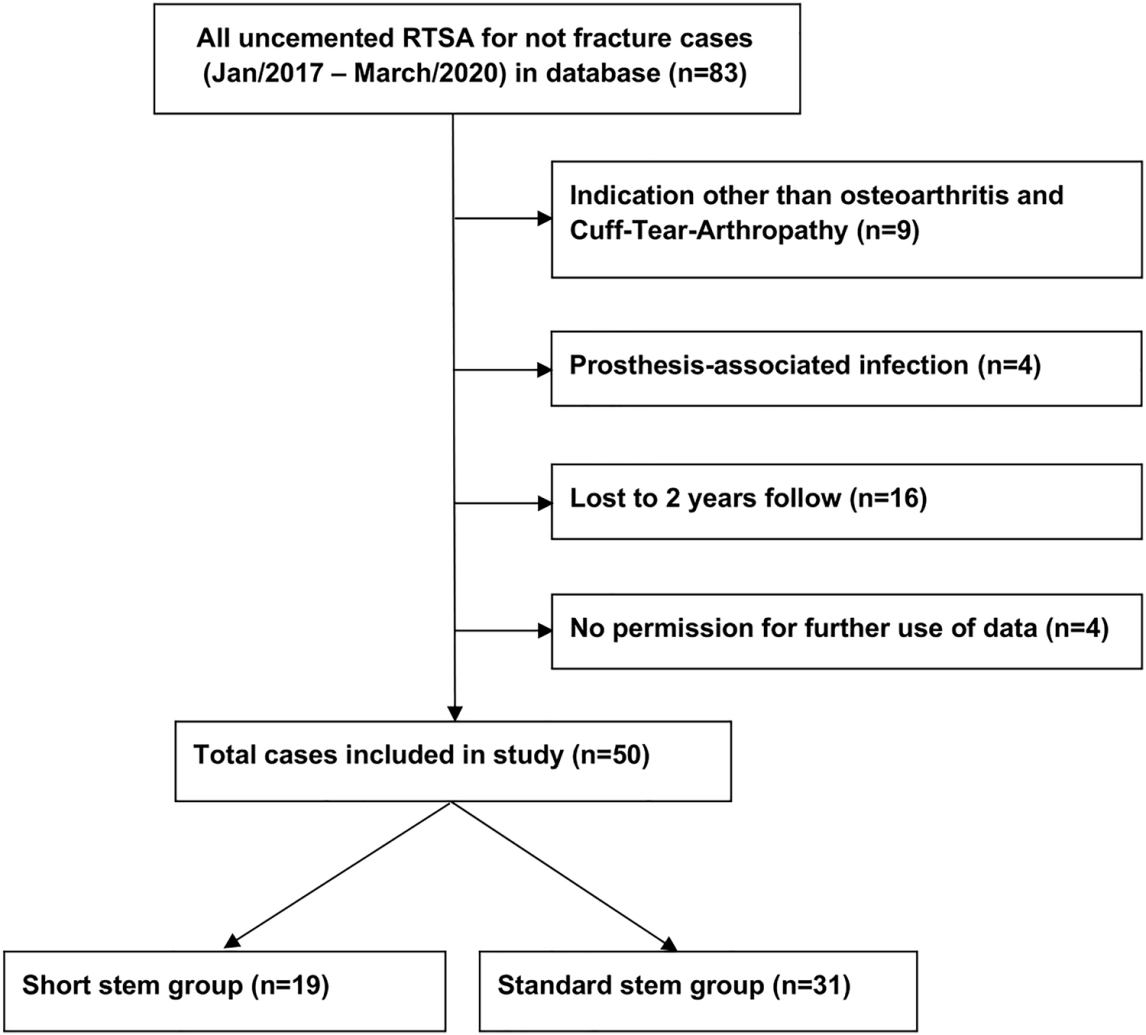
Consort diagram with patient selection and exclusion criteria

Eighty-three patients underwent primary RTSA for degenerative conditions during the study period. Four of them (5%) had a prosthesis-associated infection before the 2-year follow-up and were excluded for further study assessments. Nine patients (11%) who received prosthetic replacements for indications other than osteoarthritis or cuff tear arthropathy were excluded as well (eight avascular necrosis of the humerus and one chronic instability). A total of 16 patients were lost to 2-year follow-up and 4 individuals did not allow any further use of their data. Finally, 50 patients were included in the analysis, with 19 patients in the SHORT group (4 OA and 15 CTA) and 31 in the STANDARD group (10 OA and 21 CTA). The Consort diagram in Fig. 1 shows the selection process in detail.

All surgeries were performed by a total of four different fellowship trained shoulder surgeons using a deltopectoral approach and following the instructions of the manufacturer. Postoperative rehabilitation was the same for all patients. This included sling immobilization for 6 weeks with direct passive mobilization in internal rotation as well as functional exercises after 6 weeks and weight-bearing exercises after 12 weeks.

### Radiographic analysis

All radiographic measurements were carried out twice by two of the authors (M. K. and M.O.). For the quantitative measurements, the inter-observer variability was calculated. In case of discrepancies, all qualitative measurements were compared and discussed until consensus was reached. For the radiographic measurements, we assessed the preoperative X-rays (AP and Neer views), the direct postoperative X-rays (AP and Neer) and all remaining data on the 2-year follow-up X-rays (AP neutral, AP internal rotation, axial, Neer).

**Fig. 2:**
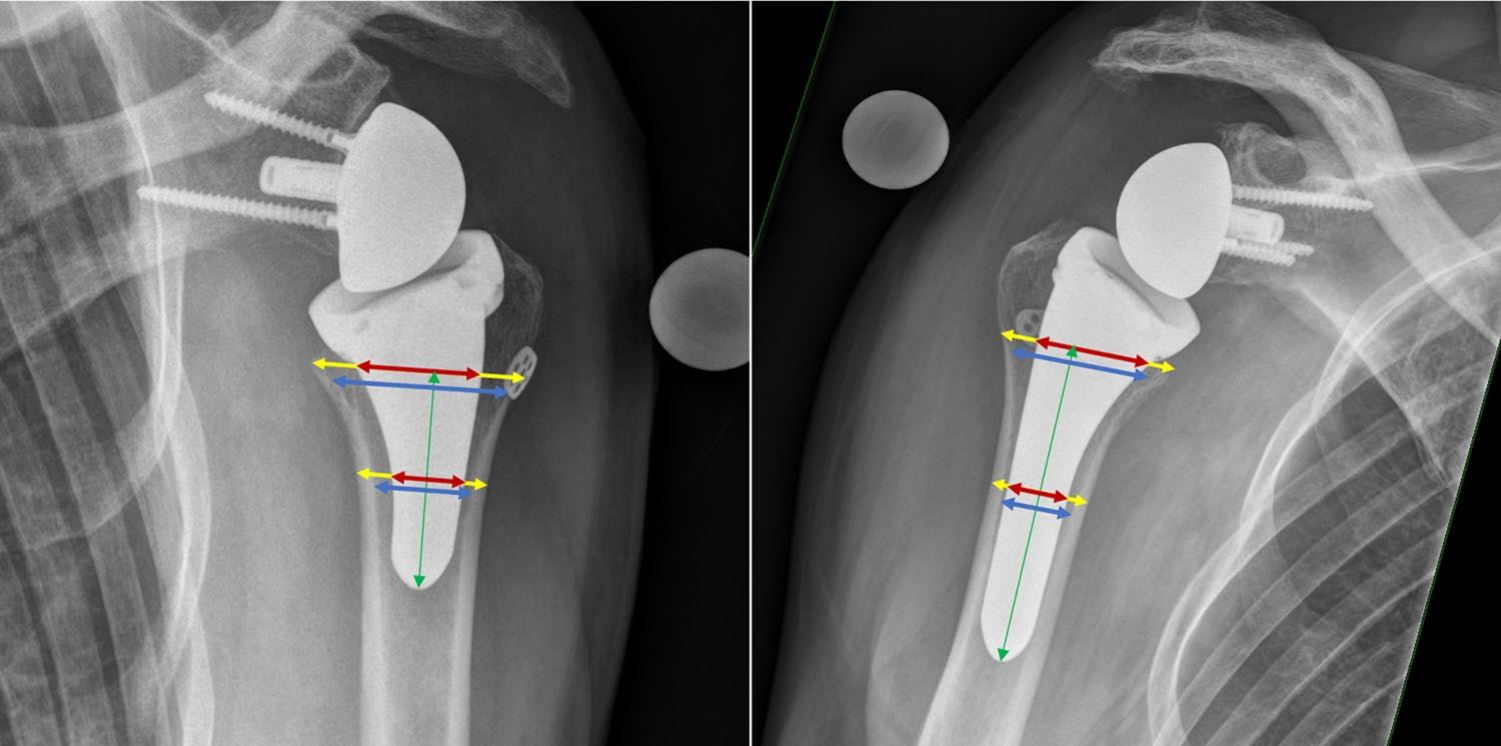
Measurements of distal and metaphyseal filling ratios in the short stem (left) and long stem (right) group. A filling ratio of stem width (red) to the distance from inner cortex boundaries (FRinnerCortices, blue) and the distance from outer cortex boundary (FRouterCortices, yellow) was calculated for the distal and metaphyseal measurements. The metaphyseal measurement was taken at the calcar level, and the distance to the stem tip was halved for the position of the distal measurement. The measurements are made perpendicular to the longitudinal axis of the prosthesis stem (green)

The bone quality was determined using the deltoid tuberosity index (DTI) [14]. The stem-to-humerus filling ratios (FR) were measured at metaphyseal and at a distal position on the anteroposterior radiograph with two different techniques following the instructions of Denard et al. [12] (measurements at the outer cortex) and Raiss et al. [13] (measurement at the inner cortex). The ratios were measured at the metaphyseal position (at the level of the calcar) at the inner and outer cortex and in the distal shaft area (half the length from the metaphyseal position to the tip of the prosthesis) position at the inner and outer cortex as described in Fig. 2.

Loosening of the prosthesis was assessed according to Sperling et al. [15] based on the occurrence of lucent lines. Numbers and locations of lucent lines were examined using the classification of Denard et al. [12]. Grading and frequency of scapular notching were recorded according to the Nerot–Sirveaux Classification [16, 17].

**Fig. 3:**
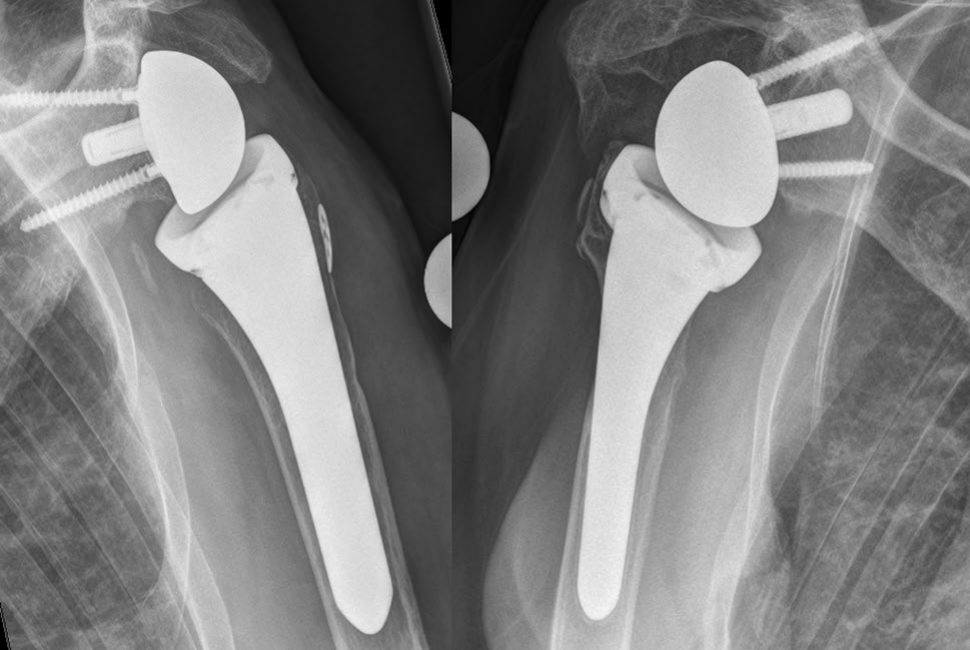
The grading system for stress shielding effect on the proximal humerus. The left side shows lateral stress shielding grade one (cortical thinning) and on the right side, there is lateral stress shielding grade 2 (complete bone loss down to the prosthesis)

Stress shielding for the humeral component, its frequency, location, and grading was examined in a slightly modified manner on the basis of the descriptions of Denard et al. [12]. Bone resorption was graded from 1 to 2 on the lateral and medial sides. Grade 1 describes a thinning of the cortex and grade 2 a complete cortical resorption down to the prosthesis. The zone classification for lucent lines and stress shielding was the same and included the zones 1 to 5 on the anteroposterior radiograph as described by Denard et al. [12]. The zone size was always set in relation to the prosthesis size and proportionally equal for the standard stem and the short stem prostheses. Examples of cases with stress shielding grade 1 (moderate) and grade 2 (severe) are shown in Fig. 3.

### Statistical analysis

All statistical calculations were performed using the open access software R (R: A language and environment for statistical computing: R Foundation for Statistical Computing, Vienna, Austria. URL http://www.R-project.org/). Means, ranges, and standard deviations were used for descriptive statistics. All measurements were carried out by two examiners and analyzed for inter-observer variability using the intraclass correlation coefficient ICC2 according to Shrout et al. [18]. For inferential statistics, *t* test and Chi-square test, i.e., Wilcoxon and Fisher exact test were used where appropriate. We applied multivariate logistic regression, ROC analysis (using “pROC” package), and recursive partitioning (using “rpart” package) to determine the best thresholds for filling ratios. The confidence level for rejecting the null hypothesis was set at 95% (*p* value < 0.05).

### Source of funding

None.

### Ethics

IRB: The Ethical Committee of the Kanton St. Gallen, Switzerland, approved the study (Ref. BASEC No. 2022-00890).

## Results

### Demographics

The total study population included 50 patients with reverse shoulder arthroplasties as primary therapy for osteoarthritis or cuff tear arthropathy (mean age = 70.6 years (44–90); 23 men: 27 women). The average age in the SHORT group was 69.2 (44–85) and did not differ significantly (*p* = 0.494) from the STANDARD group with a mean age of 71.5 (46–90). The general data are listed in Table 1; the groups showed no significant differences.

**Table 1:** Comparison of demographic data, clinical outcome data, and bone quality between short and standard stem groups

	All (*n* = 50)			
	Mean	SD	Min	Max
Age	70.6	11.5	44	90
DTI	1.44	0.14	1.19	1.72
abs CS 2 years	71.2	12.2	21	94
rel CS 2 years	95.8	17.1	23	120
SSV	86.7	13.8	45	100

	*N*	%	*N*	%
Sex (m/w)	23	46	27	54
Side (left/right)	30	60	20	40

	Short stem (*n* = 19)				Standard stem (*n* = 31)				*p* value
	Mean	SD	Min	Max	Mean	SD	Min	Max	
Age	69.2	11.5	44	85	71.5	11.6	46	90	0.494
DTI	1.46	0.14	1.29	1.72	1.43	0.14	1.19	1.72	0.514
abs CS 2 years	69.7	15.1	21	93	72.1	10.2	55	94	0.558
rel CS 2 years	91.8	22.5	23	120	98.3	12.4	74	118	0.256
SSV	86.8	15.7	45	100	86.7	12.9	45	100	0.995

	*N*	%	*N*	%	*N*	%	N	%	
Sex (m/w)	11	58	8	42	12	39	19	61	0.304
Side (left/right)	8	42	11	58	12	39	19	61	1.000

*DTI* Deltoid tuberosity index, *rel CS* relative constant score, *abs CS* absolute constant score, *SSV* subjective shoulder value

### Functional outcomes

Both groups showed similar functional results at 2-year follow-up with an absolute Constant Score of 69.7 (21–93) in SHORT and 72.1 (55–94) in STANDARD (*p* = 0.558). There was also no difference between relative Constant Score with 91.8 (23–120) in SHORT and 98.3 (74–118) in STANDARD (*p* = 0.26). Subjective shoulder values (SSV) did also not differ with 86.8% in SHORT and 86.7 in STANDARD (*p* = 0.99).

### Radiographic outcomes

The two groups did not differ with regard to bone quality (DTI) with the average value being 1.46 (1.29–1.72) in SHORT and 1.43 (1.19–1.72) in STANDARD (*p* = 0.514). Mean metaphyseal filling ratio at the outer cortex (FRoC) was 0.63 (0.57–0.75) in SHORT and 0.65 (0.50–0.82) in STANDARD (*p* = 0.216); the mean distal filling ratio at the outer cortex (dFRoC) was 0.61 (0.52–0.78) in SHORT and 0.62 (0.51–0.71) in STANDARD (*p* = 0.707). Mean metaphyseal filling ratio at the inner cortex (FRiC) was 0.68 (0.58–0.85) in SHORT and 0.71 (0.59–0.93) in STANDARD (*p* = 0.165); the mean distal filling ratio at the inner cortex (FRiC) was 0.75 (0.65–0.87) in SHORT and 0.80 (0.65–0.93) in STANDARD (*p* = 0.049). The inter-observer reliability of the DTI, distal FR, and metaphyseal FR measurements were high between the two examiners with an ICC of 0.93 (DTI), 0.81 (distal FR), and 0.84 (metaphyseal FR).

All comparisons of radiographic changes are listed in Table 2. Stress shielding was significantly more common in STANDARD (*n* = 16; 52%) than in SHORT (*n* = 4; 21%); (*p* = 0.03).The number of zones involved in stress shielding was also higher in STANDARD than in SHORT (*p* = 0.02); however, there was no significant difference in severity at the medial (*p* = 0.11) or lateral side (*p* = 0.24).

**Table 2:** Comparison of the radiographic analysis between short and standard stem groups

	Short stem (*n* = 19)				Standard stem (*n* = 31)				*p* value
	Mean	SD	Min	Max	Mean	SD	Min	Max	
DTI	1.46	0.14	1.29	1.72	1.43	0.14	1.19	1.72	0.514
dFRoC	0.61	0.07	0.52	0.78	0.62	0.06	0.51	0.71	0.707
mFRoC	0.63	0.05	0.57	0.75	0.65	0.06	0.50	0.82	0.216
dFRiC	0.75	0.07	0.65	0.87	0.80	0.07	0.65	0.93	0.049
mFRiC	0.68	0.07	0.58	0.85	0.71	0.07	0.59	0.93	0.165

	Short stem (*n* = 19)		Standard stem (*n* = 31)		*p* value
	*N*	%	*N*	%	
Stress shielding					
Yes	4	21	16	52	0.03
No	15	79	15	48	
Stress shielding zones quantity (0–5)					
0	15	79	15	48	0.02
1	3	16	3	10	
2	0	0	12	39	
3	1	5	0	0	
4	0	0	1	3	
5	0	0	0	0	
Stress shielding grade medial					
0	18	95	23	74	0.07
1	1	5	8	26	
2	0	0	0	0	
Stress shielding grade lateral					
0	15	79	18	58	0.24
1	4	21	11	35	
2	0	0	2	7	
Scapular notching					
Yes	2	11	2	7	0.61
No	17	89	29	93	
Scapular notching grade (1–4)					
0	17	90	29	93	0.61
1	2	11	2	7	
2	0	0	0	0	
> 2	0	0	0	0	
Lucent zones					
0	19	100	18	58	0.01
1	0	0	7	22	
2	0	0	2	7	
3	0	0	4	13	
4	0	0	0	0	
5	0	0	0	0	

*DTI* Deltoid tuberosity index, *dFRoC* distal filling ratio measured at the outer cortices, *mFRoC* metaphyseal filling ratio measured at the outer cortices, *dFRiC* distal filling ratio measured at the inner cortices, *mFRiC* metaphyseal filling ratio measured at the inner cortices

Scapular notching was rare with two cases in both groups graded as low (*p* = 0.61). Lucent lines were found in 13 cases in STANDARD (42%) and none in SHORT (*p* = 0.01). All were < 2 mm and thus not considered as relevant signs of loosening.

### Complications and revisions

Of the 50 patients finally included, none had any relevant clinical complication or revision surgery in the study period observed.

### Predictors of stress shielding

**Fig. 4:**
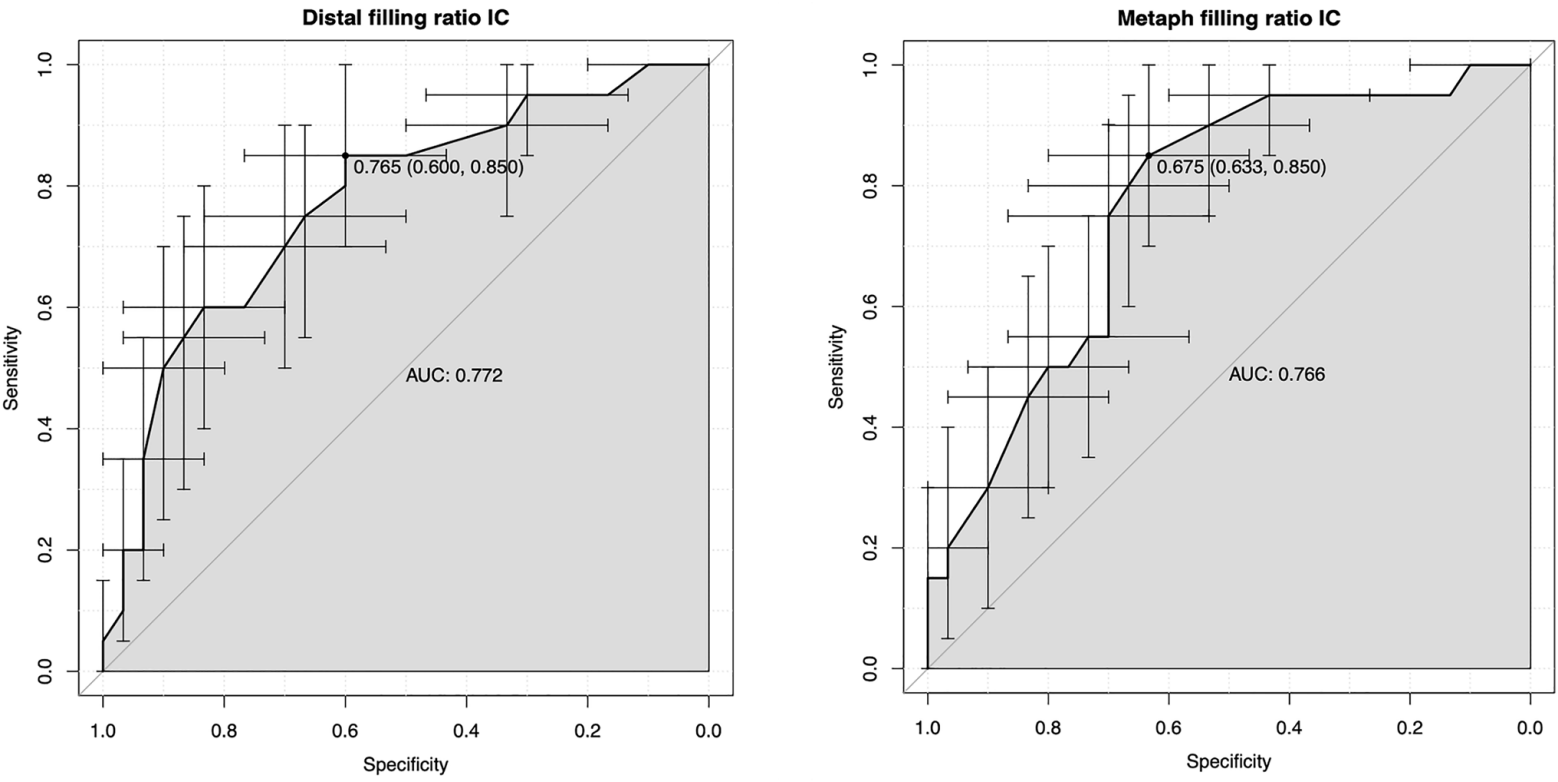
The result of the ROC is shown graphically. On the left side is the result of the distal filling ratios measured at the inner cortices (IC) and on the right side the same measurements of the metaphyseal filling ratios at the inner cortices (IC) (*ROC* receiver operating characteristic; *AUC* area under the curve)

**Fig. 5:**
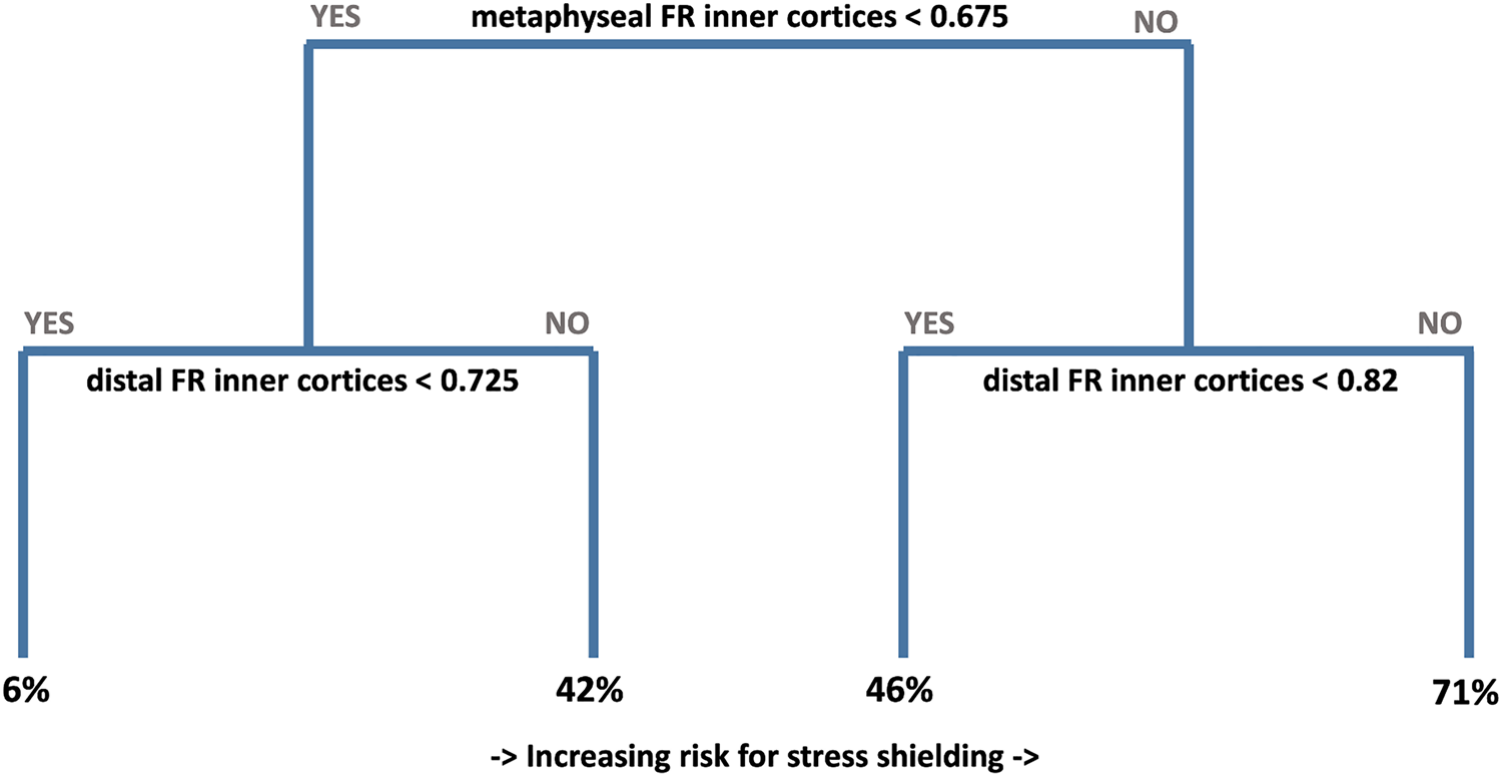
The graphic shows the effect on the occurrence of stress shielding effects when the cut-off values for the filling ratios at the inner cortices are observed. From left to right, a tenfold increase of stress shielding effects can be detected. (*FR* filling ratio)

The comparison of patients with and without stress shielding is listed in Table 3. We could not find any significant differences in age and function with a bivariate regression analysis. There was, however, a trend to lower DTI values in patients with stress shielding (1.41; 1.19–1.70) compared to those without (1.47; 1.24–1.72) (*p* = 0.099). The distal FR measured at the outer cortices were significantly higher in patients with stress shielding (0.64; 0.52–0.78) compared to those without (0.59; 0.51–0.72) (*p* = 0.006). The metaphyseal FR measured at the outer cortices were also higher in patients with stress shielding (0.67; 0.61–0.82) compared to those without (0.63; 0.50–0.76) (*p* = 0.008). We found the same results with lower *p* values for the filling ratio measurements on the inner cortices. The distal FR measured at the inner cortices were significantly higher in patients with stress shielding (0.82; 0.0.68–0.93) compared to those without (0.75; 0.65–0.92) (*p* > 0.001). The metaphyseal FR measured at the inner cortices were also higher in patients with stress shielding (0.67; 0.58–0.80) compared to those without (0.74; 0.61–0.93) (*p* > 0.001). Based on these results, we integrated all four filling ratio measures and the DTI into a multivariate regression analysis with a stepwise regression process and were able to find the filling ratios measured at the inner cortices as the only predictive variables with a *p* value < 0.05 (0.044 (metaphyseal) and 0.031 (distal)). With these measurements, we then performed a receiver operating characteristic (ROC) and found a cut-off value for the metaphyseal filling ratio of 0.68 (area under the curve = 0.77) and for the distal filling ratio of 0.77 (area under the curve = 0.77). The result of the ROC’s can be seen in Fig. 4. In addition, we performed a recursive partitioning (RP) to assess the effect of maintaining these cut-off values. This showed very similar cut-off values of 0.68 for the metaphyseal and 0.73 for the distal measurements. If neither of these two values is exceeded, the occurrence of stress shielding can be reduced to approximately 6%. Compared to this, stress shielding is about ten times more frequent when a distal value of 0.82 and a metaphyseal value of 0.68 are exceeded. The results of the RP are shown in Fig. 5.

**Table 3:** Comparison of the radiographic analysis, demographics, and functional outcomes between stress shielding and no stress shielding groups

	Stress shielding (*n* = 20)				No stress shielding (*n* = 30)				*p* value
	Mean	SD	Min	Max	Mean	SD	Min	Max	
Age	73.1	10.1	46	86	69.0	12.2	44	90	0.202
abs CS	68.3	10.5	54	87	73.1	13.1	21	94	0.150
DTI	1.410	0.120	1.190	1.700	1.470	0.150	1.240	1.720	0.099
dFRoC	0.64	0.06	0.52	0.78	0.59	0.06	0.51	0.72	0.006
mFRoC	0.67	0.05	0.61	0.82	0.63	0.06	0.50	0.76	0.008
dFRiC	0.82	0.07	0.68	0.93	0.75	0.70	0.65	0.92	0.001
mFRiC	0.67	0.06	0.58	0.80	0.74	0.07	0.61	0.93	0.001

*DTI* Deltoid tuberosity index, *rel CS* relative constant score, *abs CS* absolute constant score, *dFRoC* distal filling ratio measured at the outer cortices, *mFRoC* metaphyseal filling ratio measured at the outer cortices, *dFRiC* distal filling ratio measured at the inner cortices, *mFRiC* metaphyseal filling ratio measured at the inner cortices

## Discussion

To the best of our knowledge, this is the only study that compares follow-up data of uncemented short and standard stems from the same RTSA model regarding the effects of stem length and width on proximal humerus stress shielding over 2 years. Furthermore, it is the first publication that compares two different measurement methods for filling ratios and clears the respective ambiguities. In the literature, mainly two different methods are used for measuring filling ratios. While Denard et al. [12] measures the outer cortices, Raiss et al. [13] recommend measuring the inner cortices. In this study, we compared these two methods and could show that the measurements on the inner cortices have better predictive power for stress shielding. We recommend, therefore, the use of this measurement only in the future.

This resolves potential uncertainties in the future stress shielding research regarding which measurement method should be used. Furthermore, it seems clinically more appropriate to use the inner cortical diameter than the outer to assess the filling ratio. The outer cortical diameter includes the width of the cortex whereas the inner does not. Therefore the inner cortical measurements give a better idea of the amount of cancellous bone that may still be between the stem and the cortex. However, it is clear that both measurements are just different approaches as they may vary with different rotations and projections of the X-ray. Volumetric measurements would be the most exact method but for clinical use, the metaphyseal and distal inner cortical filling ratio seem to be good enough. We found equally good clinical results after 2 years of follow-up regardless the stem length or when stress shielding effects occurred. However, significant differences were found between the groups regarding the occurrence of stress shielding. The short stems seem to cause less stress shielding compared the standard stems. This may be due to the significantly lower distal filling ratio measured at the inner cortices which we found compared to the standard stems. Moreover, bone preserving preparation of the proximal humerus may also have an effect. The shorter the implant, the more metaphyseal load will occur after implantation. This may create a more physiological stress distribution to the metaphyseal bone leading to fewer signs of stress shielding. As expected, the filling ratios had significant effect on the occurrence of stress shielding no matter which technique was used (outer cortical or inner cortical diameter). We found a cut-off value of 0.7 (± 0.03) to lower the chances for stress shielding by ten times if not exceeded at the metaphyseal and distal measurement position. These values should be integrated in the preoperative planning to avoid stress shielding.

The relationship between increased filling ratios and stress shielding has already been demonstrated by other studies [11, 12, 19–22] and Raiss et al. postulated a cut-off value of 0.8 only for the distal measurement measured at the inner cortices in uncemented RTSA with a different short stem design [13]. This value was estimated and showed a sevenfold reduction in stress shielding cases, already an important finding. However, in our study, we were able to show that both the metaphyseal and the distal measurement have independent relevant predictive power. This is why both cut-off values should be taken into account. The expected relationship between stem length and the occurrence of stress shielding could only be found in the univariate analysis but could not be confirmed in the multivariate regression analysis. We assume that this is due to the higher filling ratios found for the standard stems, and thus stem length only has an indirect relationship to stress shielding effects when calculated together with the filling ratio. This indirect correlation could also explain the results of Denard et al. who found a correlation between longer stems and stress shielding [9]. We also suspect an indirect relationship between poor bone quality and a higher rate of stress shielding; however, this was only a trend and not statistically significant in this study. The reason for this may be bivariate: first, there is less bone to start with and thus it is prone to an earlier occurrence of stress shielding; second, surgeons could choose a wider stem—thus a larger filling ratio—in case of poor bone quality, to prevent subsidence, loosening or malalignment. During implantation, a compromise must be made between a large implant for good anchorage of the stem and the smallest possible implant to prevent stress shielding.

In addition to the radiographic findings, our study is in accordance with all other studies on this topic: no loss of functional outcome due to the occurrence of stress shielding could so far be detected [12, 23–25].

## Limitations

The data were collected prospectively but analyzed retrospectively. Furthermore, the study population includes a relatively small number of cases. However, it was not underpowered to answer the main hypothesis. The group size is similar to the comparable literature, but not sufficient to perform a meaningful subgroup analysis.

The study was underpowered to determine the effect of bone quality on stress shielding. However, the bone quality did not differ between the two groups which is a strength of our findings and proves no selection bias. DTI measurements showed a trend for more frequent stress shielding effects with poorer bone quality (although not statistically significant).

Two years of follow-up is certainly not enough to draw final conclusions about the relevance of stress shielding effects for long-term outcome and revision surgeries.

The surgeries were performed by four different fellowship trained surgeons. Even though the technique is the same by all surgeons in our institution, differences in implantation could lead to bias.

## Conclusion

We found no clinical difference between uncemented short stems and standard stems in RTSA for degenerative indications. Also the occurrence of stress shielding had no influence on the clinical outcome after 2 years. Short stems seem to cause less stress shielding than longer standard stems; however, the effect of higher filling ratios in standard stems may outweigh the effect of stem length. We found a ten times smaller rate of stress shielding, regardless the stem length, if the filling ratios (measured at the inner cortical diameter at the metaphysis and at the distal stem) were less than 0.7 (± 0.03). This finding should be included for the future planning of such prostheses. Moreover, according to our results, the filling ratio should be calculated using the inner cortical diameter.
